# Computer aided self-regulation learning and cognitive training improve generalization ability of patients with poststroke cognitive impairment

**DOI:** 10.1038/s41598-021-03620-1

**Published:** 2021-12-17

**Authors:** He Youze, Yang Ting, Bao Yaqi, Xiao Tianshen, Wu Tiecheng, Wu Jingsong

**Affiliations:** 1 College of Rehabilitation Medicine, Fujian University of Traditional Chinese Medicine, Fuzhou, China; 2 Fujian Collaborative Innovation Center for Rehabilitation Technology, Fuzhou, China

**Keywords:** Psychiatric disorders, Neuroscience, Cognitive neuroscience, Diseases of the nervous system, Regeneration and repair in the nervous system

## Abstract

Emerging studies suggest the application of self-regulation learning (SRL) to improve generalization abilities in poststroke patients. SRL has been proposed to have an added effect on computer-aided cognitive training (CACT). This study aimed to examine the efficacy of an intervention combining computer-aided SRL (CA-SRL) training and CACT for generalization abilities and cognitive function in patients with poststroke cognitive impairment (PSCI). A total of 75 patients recruited from a rehabilitation centre were randomly assigned to a CA-SRL group, demonstration learning (DL) group and traditional learning (TL) group. Finally, 72 patients were included in the analysis. Over 3 weeks, the patients in these three groups underwent CA-SRL or DL training combined with cognitive training. After the intervention, all outcomes significantly improved (*P* < 0.05). The CA-SRL group showed better improvements in all trained tasks among the groups, especially in "wash the dishes" and “change the bed”. The results of the analysis for generalization abilities showed that CA-SRL group patients obtained the highest scores among the groups in untrained tasks. The mean changes in the Montreal Cognitive Assessment in both the CA-SRL and TL groups were significantly higher than those in the DL group (*P* < 0.001, *P* = 0.002) after adjusting for education level and Lawton Instrumental ADL Scale score. In general, the combination of CA-SRL and CACT is effective for PSCI patients and has a better effect on promoting skill generalization from cognitive gains than traditional training.

## Introduction

Poststroke cognitive impairment (PSCI) is a commonly observed dysfunction in patients with stroke^1^, and 76.3% of subacute poststroke patients and 67.3% of chronic poststroke patients have been reported to have cognitive impairments in at least one cognitive domain, such as attention, memory, executive function and perceptual disorders^2,3^. Moreover, impairments in cognitive functions negatively impact these patients’ activities of daily living (ADL) and reduce their abilities to reacquire motor and functional skills, leading to a decrease in quality of life^4–6^.

Cognitive training is one of the most widely used rehabilitation strategies for PSCI patients, and its effectiveness and safety for recovering cognitive functions has been demonstrated^7,8^. However, some previous studies revealed that cognitive function improvements from cognitive training were not generalizable to ADL^9–11^. The possible reason for this phenomenon may be the training scenes and contents unrelated to normal activities of daily life^12^. Moreover, it might be correlated with the injury to brain regions that caused impairments in learning and relearning abilities and thus limited generalization from cognitive functions to ADL^13^.

Self-regulation learning (SRL) training, which includes three steps (decomposition step, self-calibration and mental imagery), is a widely used method to improve generalization abilities and daily task performance in poststroke patients^14–16^. SRL training has been demonstrated to encourage individuals to adapt task strategies to the tasking environment^17^, but the transfer effect of SRL training on cognitive functions, such as memory, was minimal^18^. Moreover, the manual training involved in SRL has relatively high requirements, such as reliance on the knowledge and experience of therapists, limitations related to training sites and training tools, and costs associated with therapists’ time in preparing training contents.

The development of computer-aided technology in recent years has provided a new solution for the rehabilitation of poststroke patients. Compared with traditional one-on-one training, computer-aided training can provide various training contents in different forms based on cost-effectiveness^19^. Many systematic review and meta-analysis studies have demonstrated the practical efficacy of computer-aided cognitive training (CACT) in improving the cognitive functions of poststroke patients^20,21^. Combining cognitive training with SRL training to form a new computer-aided strategy for the rehabilitation of poststroke patients may help improve patients' cognitive functions and promote the generalization of their skills.

This study designed the first Chinese computer-aided SRL (CA-SRL) training programme and combined the new programme with CACT to form a comprehensive intervention scheme for PSCI patients. We conducted a randomized controlled trial to answer the following questions: (1) Is this intervention combining CA-SRL and CACT effective for improving the generalization abilities and cognitive functions of PSCI patients? (2) Does CA-SRL training better facilitate generalization of cognitive gains to ADL than traditional demonstration learning and cognitive training?

## Methods

### Study design

This study was a single-blinded randomized controlled trial for poststroke patients with cognitive impairment. The protocol for this trial was approved by the Ethics Committee of the Rehabilitation Hospital Affiliated to Fujian University of Traditional Chinese Medicine (Approved number: 2017YJS-004-01) in 2017. This study was performed in accordance with the guidelines of the Declaration of Helsinki, and informed consent was obtained from all participants and/or their legal guardians before screening. The details of this trial were also registered in the China Clinical Trial Registry (Registration number: ChiCTR-INR-17013042; Registration date: 19/10/2017).

### Participants

Eligible participants were recruited from the Rehabilitation Hospital Affiliated with Fujian University of Traditional Chinese Medicine. All participants included met the following inclusion criteria: (1) met the diagnostic criteria for stroke according to the principles of 2010 Chinese guidelines for the management of hypertension^22^ with confirmation by computed tomography or magnetic resonance imaging; (2) experienced first-ever stroke and were within one year poststroke; (3) were aged between 45 and 80 years; (4) had an education level of primary school or above; (5) had cognitive impairment identified by the Fuzhou version of the Montreal Cognitive Assessment^23^ with scores between 10 and 26 points; (6) experienced cognitive impairment after stroke with exclusion of other causes; (7) were conscious and had stable vital signs; (8) could understand the execution of computer-aided training; and (9) provided informed consent themselves, or consent was provided by their legal guardians.

Exclusion criteria: (1) severe visual or hearing impairment or mental disorders due to their potential impacts on cognitive examination; (2) a history of mental retardation and dementia before the occurrence of stroke; (3) severe unilateral spatial neglect; (4) depressive symptoms (Hamilton Depression Scale score > 7)^24^; (5) severe heart disease, liver failure, renal failure, malignant tumour, gastrointestinal bleeding or other diseases; and (6) inclusion in other clinical trials that would affect outcome assessments in our study.

### Procedures

**Figure 1:**
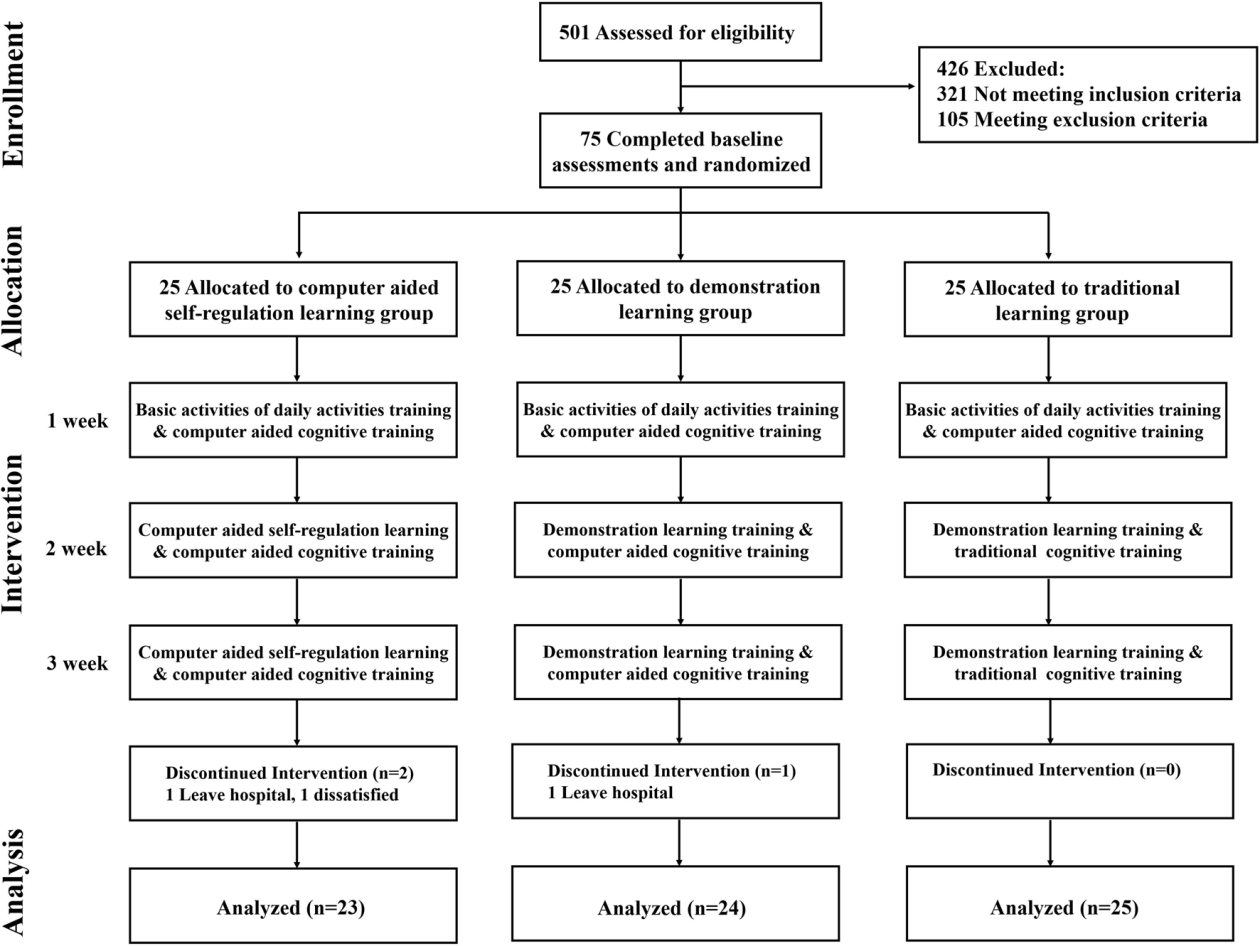
Flowchart of this study showing the procedure of enrollment, allocation, intervention, and analysis.

After obtaining participants’ written informed consent forms and screening for inclusion and exclusion, all included patients were randomly assigned to the computer-aided self-regulation learning group, demonstration learning group or traditional learning group at a ratio of 1:1:1. Randomization was conducted by an independent researcher who did not participate in the intervention and outcome assessments of our study. The randomized sequence was generated using simple randomization in SPSS 24.0 statistical analysis software (SPSS, Inc., Chicago, IL). After completing baseline assessments, the independent researcher informed these patients about the results of their group allocation via telephone. Due to the full explanation of interventional contents to eligible participants in the informed consent form, blinding of the included patients was not possible. Outcome assessors, data entry personnel and statistical analysts were blinded to avoid potential bias. Meanwhile, to ensure the quality of training, all interventions were administered by occupational therapists with more than three years of working experience in poststroke rehabilitation who did not participate in patient enrolment and outcome assessments. The schedule and contents of the interventions in this study are shown in Fig. 1.

### Intervention

#### Computer-aided self-regulation learning (CA-SRL) group

Patients in the CA-SRL group received CA-SRL training, CACT and basic ADL training. In the first week, basic ADL training was conducted during five sessions, with one hour per session, which was the basis of SRL training. Through the first week of training, patients were able to understand the contents involved in SRL training, which prevented the potential effect of failure to comprehend the training contents, which would limit the efficacy of CA-SRL training. The contents of CA-SRL (including ten tasks related to ADL) were carried out for one hour per session five times per week in the next 2 weeks. In addition, CACT was carried out throughout the 3 weeks for 30 min each session.

#### Demonstration learning (DL) group

Training tasks in the DL group were also divided into three parts, and the details of each part were relayed to the patients for comparison for error-free calibration^25,26^. In the first week, patients received the same basic ADL training with the same training dose. Unlike the CA-SRL group, these ten training tasks were conducted in a demonstration learning manner with the same training schedule. Moreover, CACT was also conducted throughout these 3 weeks for 30 min each session.

#### Traditional learning (TL) group

Similar to the training schedule in the DL group, the patients in the TL group received traditional cognitive training with the same training dose rather than CACT. In addition, other training parts (basic ADL training and DL training) were the same as those in the schedule for the DL group.

### Interventional contents

#### Computer-aided self-regulation learning training

The learning tasks for CA-SRL consist of three parts: step decomposition, self-calibration and mental imagery^27^. Consistent with our design, during CA-SRL, the process of patients’ task learning was recorded by cameras, and then the videos were displayed to allow self-reflection on their task performance^14,28^. Moreover, CA-SRL training consists of ten daily tasks^29^, including folding laundry, visiting a doctor, making a bed, tidying a table after a meal, frying vegetables with meat, preparing fruit, sweeping a floor, washing dishes, changing bedding and taking medication. During CA-SRL training, therapists guided the patients according to the preprogrammed instructions to understand the task's actions, self-reflect and think about the solution, and finally complete each task. In the second week, the patients were trained in five daily tasks: folding laundry, visiting a doctor, making a bed, tidying a table after a meal, and frying vegetables with meat. Then, in the third week, the patients were trained in the other five tasks: washing dishes, changing bedding, preparing fruit, taking medication and sweeping a floor.

#### Computer-aided cognitive training

In this study, the contents of CACT were developed by the cognitive rehabilitation research team of Hong Kong Polytechnic University, Hong Kong University and Fujian University of Traditional Chinese Medicine. The two parts of CACT were named the basic cognitive module and cognitive application module. The primary cognitive module addressed simple reaction time, visual perception, visual attention, visual choice, sustained attention, working memory, a maze, one mind for two purposes, psychological rotation and auditory choice, which aimed to improve specific cognitive functions. In addition, the cognitive application module consisted of four parts: computer application training, memory application training, logic ability training and attention application training. These training items are intended to integrate cognitive training contents into a daily life context to improve comprehensive cognitive abilities. As designed, professional therapists were appointed to arrange the training contents according to the actual situations of the patients.

#### Demonstration learning training

DL training consisted of three steps: step decomposition, error-free calibration and repetitive training^25^. Task manipulation (the main steps and the required tools and sequence) was relayed in detail to the patients for comparison for error-free calibration. Before completion of all tasks, the therapists provided patients with sufficient demonstrations and corrected inaccurate actions in a timely manner. Then, the patients were expected to practice this item repeatedly until completion of the task independently^30^. All training contents were consistent with CA-SRL contents.

#### Basic ADL training

Professional therapists conducted the basic activities of daily living training based on the Code of Practice for Common Rehabilitation Therapy Techniques (2012 edition). The training contents included clothing, decoration, eating, bed and chair transfer, using the toilet, bathing, and going up and down stairs. According to the conditions and performances of the patients, occupational therapists evaluated the patients’ activities and then prepared the corresponding tools, such as clothes for dressing and spoons for eating. Through observations and evaluations, the missing components of their actions were relayed to the patients, and then these actions were practiced repeatedly.

#### Traditional cognitive training

Based on the Code of Practice for Common Rehabilitation Therapy Techniques (2012 edition), the contents of traditional cognitive training were composed of attention, memory, calculation and discrimination training. Therapists were appointed to conduct traditional cognitive training for the patients according to their cognitive assessment results.

### Outcome assessment

The primary outcomes were daily living task assessment^29^ and Montreal Cognitive Assessment (MoCA), Fuzhou version^23^. The Daily Living Task Assessment was applied to assess the patients’ relearning and generalization abilities (reliability: 0.89–0.98, kappa value: 0.54–0.72)^29^. In the relearning test, the previous five trained tasks (wash the dishes, change bedding, prepare fruit, take medication and sweep the floor) were tested twice at baseline and after the intervention. Another five untrained daily tasks (including cook and steam fish, put clothes on a hanger, use the telephone, use the canteen and clean the bathroom) were conducted to assess generalization abilities, which were tested only once after intervention^29^. These subtests were both scored from 1 (total assistance, unable to perform 25% or more of the task) to 7 (complete independence, able to perform the task in a timely and safe manner)^29^. A higher score corresponds to greater independence. The MoCA was used to assess global cognitive function.

The secondary outcomes were the Modified Barthel Index (MBI)^31^, Lawton Instrumental Activity of Daily Living Scale (IADLs)^32,33^ and Simplified Fugl-Meyer Assessment (FMA)^34^. The MBI and IADLs were applied to evaluate patients' basic ADL and instrumental ADL abilities, respectively. The FMA was used to assess the motor functions of poststroke patients.

### Statistical analysis

Except for evaluating generalization abilities after the intervention, other outcomes were all assessed at baseline and 3 weeks postintervention. All data included in the analysis were calculated by IBM SPSS Version 24.0 software. Categorical variables were expressed in numbers by several different categories using chi-square (χ^2^) tests. Continuous variables were presented as the mean (SD) and calculated according to their distribution. If the continuous variables were normally distributed, a one-way analysis of covariance (ANCOVA) was applied to compare differences between the three groups. Otherwise, the Kruskal–Wallis rank-sum test was applied.

In our analysis, the Per Protocol Set was applied, and only the patients who completed the whole trial were included. Moreover, to clarify the efficacy of the intervention, differences between baseline and postintervention were compared by a paired t test or the Wilcoxon signed-rank test. Due to significant differences in education levels and IADLs scores across the three groups at baseline, these factors were added as covariates in ANCOVA for multiple comparisons. *P*-values below 0.05 (two-tailed) were considered statistically significant.

## Results

All PSCI patients were recruited from the Department of Neurological Rehabilitation, the Rehabilitation Hospital Affiliated to Fujian University of Traditional Chinese Medicine. A total of 501 patients were screened, 321 of whom did not meet the inclusion criteria, while 105 met the exclusion criteria. Therefore, 75 PSCI patients were finally included in this study. (See Fig. 1).

During the intervention, two patients discontinued the programme and were discharged from the hospital due to economic reasons. Another patient discontinued th programme because of dissatisfaction with these tests. A total of 72 patients (CA-SRL group: n = 23; DL group: n = 24; TL group: n = 25) completed all interventions, and no substantial harm to participants or severe adverse events occurred during the trial.

The demographic and baseline characteristics of these three groups are presented in Table 1. Except for education levels and IADLs, no significant differences in age, sex, stroke duration, stroke location, the type of stroke or five daily living tasks were found at the baseline evaluation.

**Table 1:** Demographic and baseline characteristics of three groups [mean (SD)/M (P25, P75)].

Variables	CA-SRL group (n = 23)	DL group (n = 24)	TL group (n = 25)	F/Z/χ^2^	*P*-value
Age, year^b^	57 (51, 65)	57 (48.25, 64)	58 (51.5, 66)	0.592	0.744
Gender (men/women, n)^c^	19/6	18/7	19/6	0.141	0.932
Education, year^b^	9 (9, 12)	9 (9, 12)	7 (5, 11)	10.923	**0.004**
Months after stroke^b^	2 (1, 7)	2 (1, 6.5)	1 (1, 3)	3.960	0.138
Location of stroke (Left/Right, n)^c^	19/6	17/8	17/8	0.773	0.537
Types (ischemic/hemorrhagic, n)^c^	15/10	18/7	17/8	0.657	0.551
MoCA^a^	19.22 (4.22)	19.17 (4.57)	16.60 (6.04)	2.167	0.122
MBI^a^	61.39 (19.71)	59.88 (17.24)	51.04 (27.08)	1.594	0.211
IADLs^b^	6 (5, 11)	8 (5.25, 9)	4 (4, 7)	8.538	**0.014**
**FMA**					
Upper limbs^b^	14 (10, 28)	19 (7, 37.5)	25 (6, 49)	0.547	0.761
Lower limbs^b^	22 (18, 27)	18.5 (16, 24.5)	18 (10, 28)	2.365	0.307
**Five daily living tasks**					
Wash the dishes^b^	4 (3, 5)	4 (4,5)	3 (4,4.5)	2.114	0.348
Change the beds^b^	3 (2, 3)	3 (3, 3)	3 (2, 3)	0.249	0.883
Sweep the floor^b^	4 (3, 5)	4 (3, 5)	4 (3, 4)	0.792	0.673
Take medication^b^	3 (3, 4)	3.5 (3, 4)	3 (3, 4)	0.602	0.740
Prepare fruit^b^	3 (3, 4)	4 (3, 4)	3 (2, 4)	3.499	0.174

^a^Calculated by ANOVA.

^b^Calculated by Kruskal–Wallis rank sum test.

^c^Calculated by Chi-square test.

Significant values are in [bold].

Our results showed that all outcomes significantly improved after the intervention (*P* < 0.05) (see Table 2). Statistically significant differences in MoCA and IADLs scores and relearning ability in five tasks (*P* < 0.001) were identified among the groups. Further multiple comparisons showed that the improvements in MoCA scores in both the CA-SRL and TL groups were significantly greater than those in the DL group (*P* < 0.001, *P* = 0.002, respectively). In comparing IADLs, improvements in the CA-SRL group were better than those in the TL group (*P* = 0.033). The results for relearning ability showed that improvements in all five trained tasks in the CA-SRL group were significantly better than those in the DL group (*P* < 0.05). In addition, the CA-SRL group also showed better improvements in “wash the dishes” (*P* = 0.046) and “change the bed” *(P* = 0.016) than the TL group. In the comparison between the DL and TL groups for these trained tasks, only one task (taking medication) showed that the improvement in the TL group was more significant than that in the DL group (*P* < 0.001). However, no significant differences in MBI and FMA scores were found. (See Table 3).

**Table 2:** Comparison of outcomes before and after intervention [mean(SD)].

Variables	CA-SRL group (n = 23)		DL group (n = 24)		TL group (n = 25)	
	Pre	Post	Pre	Post	Pre	Post
MoCA	19.36 (4.32)	23.22 (3.69)^a^	19.08 (4.49)	20.42 (4.76)^a^	16.60 (6.04)	19.92 (5.78)^a^
MBI	60.24 (20.26)	79.52 (13.68)^a^	58.28 (18.66)	75.63 (14.61)^a^	51.04 (27.08)	67.64 (24.51)^a^
IADLs	7.96 (3.05)	11.65 (3.92)^a^	7.68 (2.93)	10.13 (2.97)^a^	5.44 (3.71)	6.96 (5.73)^a^
**FMA**						
Upper limbs	21.80 (16.62)	27.00 (14.32)^a^	22.12 (14.73)	26.38 (18.54)^a^	27.36 (21.08)	34.72 (21.81)^a^
Lower limbs	21.60 (6.62)	24.04 (5.20)^a^	19.28 (6.07)	20.75 (6.71)^a^	18.12 (9.66)	20.80 (8.54)^a^
**Relearning ability**						
Wash the dishes	4.12 (0.88)	6.04 (0.98)^a^	4.16 (0.75)	5.33 (0.92)^a^	3.80 (0.91)	5.00 (1.00)^a^
Change the beds	2.88 (0.73)	4.52 (0.79)^a^	2.92 (0.64)	3.96 (0.69)^a^	2.84 (0.80)	4.00 (0.65)^a^
Sweep the floor	3.96 (0.89)	5.65 (1.19)^a^	3.88 (1.05)	4.79 (1.06)^a^	3.72 (0.94)	5.12 (0.97)^a^
Take medication	3.48 (0.59)	4.91 (0.73)^a^	3.68 (0.85)	4.46 (0.83)^a^	3.60 (0.76)	5.48 (1.33)^a^
Prepare fruit	3.48 (1.05)	5.04 (1.02)^a^	3.76 (0.66)	4.42 (0.83)^a^	3.28 (1.10)	4.48 (0.92)^a^

^**a**^**:** Comparison between Pre- and Post-intervention; *P*-value < 0.05. These results in this table showed the significant effect of these interventions (CA-SRL, DL and TL) in improving cognitive function, quality of life, activities of daily living, motor functions, and relearning abilities.

**Table 3:** Comparison of mean changes of outcomes after intervention [mean (SD)].

Variables	CA-SRL group (n = 23)	DL group (n = 24)	TL group (n = 25)	*F*-value	Differences *P*-value	CA-SRL versus DL *P*-value	DL versus TL *P*-value	CA-SRL versus TL *P*-value
MoCA	4.00 (2.09)	1.25 (1.26)	3.32 (1.70)	19.706	** < 0.001**	** < 0.001**	**0.002**	0.100
MBI	18.13 (12.64)	15.75 (10.91)	16.60 (15.41)	0.507	0.605			
IADLs	3.84 (0.70)	2.67 (0.70)	1.15 (0.71)	3.414	**0.017**	0.685	0.452	**0.033**
**FMA**								
Upper limbs	7.12 (2.06)	3.31 (2.06)	7.66 (2.11)	1.273	0.287			
Lower limbs	2.26 (4.04)	1.42 (2.52)	2.68 (5.92)	0.221	0.802			
**Relearning ability**								
Wash the dishes	1.89 (0.19)	1.18 (0.19)	1.22 (0.20)	4.387	**0.016**	**0.029**	1	**0.046**
Change the bed	1.65 (0.14)	1.04 (0.14)	1.07 (0.14)	6.453	**0.003**	**0.006**	1	**0.016**
Sweep the floor	1.70 (1.06)	0.88 (0.74)	1.40 (1.00)	3.902	**0.025**	**0.021**	0.423	0.818
Take medication	1.43 (0.84)	0.75 (0.53)	1.88 (1.20)	11.384	** < 0.001**	**0.027**	** < 0.001**	0.077
Prepare fruit	1.61 (0.94)	0.63 (0.58)	1.20 (0.96)	8.451	**0.001**	** < 0.001**	0.271	0.117

The table showed the comparison results of mean changes of MoCA, MBI, IADLs, FMA and relearning abilities in Analysis of Covariance after Adjusting education year and scores of IADLs.

Significant values are in [bold].

In the assessment of generalization ability, significant differences were noted among the three groups in the five untrained tasks (*P* < 0.05). The comparison results showed that the scores for the tasks "go to the canteen", "put clothes on hanger" and "use the telephone" in the CA-SRL group were significantly better than those in the DL group (*P* = 0.001, *P* = 0.005, *P* = 0.007, respectively). Moreover, the CA-SRL group also showed significantly higher scores than the TL group in the tasks "go to the canteen" *(P* = 0.002), "clean the bathroom" (*P* < 0.001) and "cook and steam fish" (*P* = 0.001). (See Table 4).

**Table 4:** Comparison of generalization ability assessment between groups [mean (SD)].

Variables	CA-SRL group (n = 23)	DL group (n = 24)	TL group (n = 25)	*F*-value	Differences *P*-value	CA-SRL versus DL *P*-value	DL versus TL *P*-value	CA-SRL versus TL *P*-value
Go to the canteen	4.87 (0.82)	3.96 (0.81)	3.76 (0.83)	9.779	** < 0.001**	**0.001**	1	**0.002**
Put clothes on hanger	5.26 (0.86)	4.50 (0.78)	4.44 (1.04)	5.446	**0.006**	**0.005**	0.747	0.190
Clean the bathroom	4.74 (0.69)	4.05 (0.61)	3.56 (0.82)	11.442	** < 0.001**	0.078	0.064	** < 0.001**
Cook and steam fish	5.00 (0.74)	4.54 (0.72)	3.88 (0.88)	7.531	**0.001**	0.156	0.152	**0.001**
Use the telephone	5.65 (1.23)	4.75 (0.85)	4.72 (0.94)	5.513	**0.006**	**0.007**	1	0.071

The group differences were compared after adjusting education year and scores of IADLs.

Significant values are in [bold].

## Discussion

To our knowledge, this is the first study to observe the efficacy of combining CA-SRL and CACT for PSCI patients for their generalization abilities and cognitive function. Our results revealed that the combined strategy resulted in significant improvements in generalization abilities and cognitive function compared with baseline findings and those achieved with DL or TL training. We also found that patients in the CA-SRL group achieved higher scores in five untrained tasks after CA-SRL training, illustrating the effect of CA-SRL in promoting skill generalization from cognitive gains.

We found that these three rehabilitation strategies significantly improved global cognitive function, ADL execution, motor functions and relearning ability after the 3-week intervention compared with the baseline findings. Moreover, the between-group analysis indicated that the combined strategy (CA-SRL + CACT) had a better effect on enhancing performance in most of the trained tasks and half of the untrained tasks compared to demonstration learning training, which might reflect the benefits of CA-SRL training. Because the SRL strategy provided opportunities to make errors and learn to recognize and self-correct these errors^35^, demonstration learning (also named errorless learning) focused on motion imitations and required a high level of support from the therapist to prevent errors and promote errorless performance in daily situations^36^. Thus, patients in the CA-SRL group had more opportunities to discover their faults in task performance and try to determine the appropriate solutions to solve and correct the faults. At the same time, the patients submitted to demonstration learning in the DL or TL group had difficulty forming self-awareness of self-correction^37^, which is consistent with other observations in a previous study. Research demonstrated significantly fewer errors and greater behavioural competency after SRL training than errorless training in patients with traumatic brain injury (TBI), which demonstrated the greater efficacy in enhancing skills generalization abilities on tasks related to training^26^ and thus provided evidence for the practical efficacy of the combined strategy (CA-SRL + CACT) in improving generalization abilities for transferring skills to untrained tasks.

In comparing the generalization abilities of PSCI patients between the DL and TL groups, no significant difference in the scores for these five untrained tasks were found, which may be the result of demonstration learning, which lacked thinking and reflection on tasking execution during the training process^12^. However, higher scores on five untrained tasks were found in the CA-SRL group, which provided further evidence for the efficacy of the combined strategy (CA-SRL + CACT) in promoting skill generalization. In other words, these scores revealed the better effect of CA-SRL than manual training in generalizing cognitive gains to task performance.

In the analysis of relearning ability, we also found better improvement in “take medication” in the TL group (mean change = 1.88) than in the CA-SRL group (mean change = 1.43) or DL group (mean change = 0.75). This task is a simple sequence and requires comparatively less upper extremity motor function than other tasks. Therefore, the improvement in this task may be associated with changes in motor functions of the upper limb. Although no significant difference was identified, the mean changes in the upper limb FMA score in the TL group were higher than those in the CA-SRL and DL groups, which might provide a possible explanation for this result. In addition, the therapists used some additional tools during traditional cognitive training, such as pencils, paper and cards, to guide training execution. Some traditional training tasks, such as writing, using fingers to recognize cards, and folding paper, can improve upper limb motor functions. In another aspect, a previous study declared that the efficacy of CACT in improving motor function was uncertain compared with that of traditional cognitive training^38^. Therefore, this might be a reasonable explanation for the significant "take medication" task in the TL group.

Additionally, when comparing changes in global cognitive function among these groups, our results showed that both the interventions in the CA-SRL and TL groups had a more significant effect on global cognitive function than the DL intervention, but no significant difference was foun between them. Although CACT could significantly improve the cognitive function of patients with cognitive impairment after brain injury, its effect was not significantly different from or even lower than that of traditional cognitive training^11,39–41^. Cognitive intervention mainly concentrated in one cognitive domain has been proposed to be more effective than multiple domains for stroke patients^11^. In our study, the contents of CACT involved multiple cognitive domains. In contrast, traditional cognitive training was guided by professional therapists to ensure the motivation of PSCI patients to complete complex cognitive tasks. This would make patients obtain more benefits and improvements in cognitive function in some targeted cognitive domains^42^. Moreover, although there was no significant difference between the CA-SRL and TL groups, the mean changes in the MoCA score in the CA-SRL group were the highest among these groups. The more significant effect may be the benefits of CA-SRL training, which motivated patients to use the problem-solving method with active self-reflection and repetitive practice to complete their tasks to activate more changes in cognitive functions^27^. Therefore, it could be believed that the combined strategy (CA-SRL + CACT) was also influential in improving global cognitive function.

This study had several limitations. First, whether patients were actively engaged in each training session was impossible to control in this study. Suppose patients were trained with low enthusiasm and effort, which may increase the between-group variability of results and then lower the significance of the training effect of CA-SRL. Second, we did not consider the patients' personal experience with similar training contents, which may also affect our results to some extent. Future studies could focus more on these points to reduce the impacts of these mixed factors.

## Conclusion

The combination strategy of CA-SRL and CACT is an effective tool for improving the generalization abilities, cognitive function and motor functions of PSCI patients. The efficacy of combining CA-SRL with CACT on skill generalization improvements is better than that of traditional strategies of training. However, its effect on global cognitive function is similar to that of traditional training. Given the advantages of computer-aided technology, including low costs, high availability, and no limitation by the treatment environment, the combined strategy is worth applying in rehabilitation interventions for PSCI patients.
